# Phage Biodiversity in Artisanal Cheese Wheys Reflects the Complexity of the Fermentation Process

**DOI:** 10.3390/v9030045

**Published:** 2017-03-16

**Authors:** Jennifer Mahony, Angelo Moscarelli, Philip Kelleher, Gabriele A. Lugli, Marco Ventura, Luca Settanni, Douwe van Sinderen

**Affiliations:** 1 School of Microbiology, University College Cork, T12 YT20 Cork, Ireland; j.mahony@ucc.ie (J.M.); angelo.moscarelli@gmail.com (A.M.); p.r.kelleher@umail.ucc.ie (P.K.); 2 Alimentary Pharmabiotic Centre, University College Cork, T12 YT20 Cork, Ireland; 3 Department of Agricultural and Forest Science, University of Palermo, 90128 Palermo, Italy; luca.settanni@unipa.it; 4 Laboratory of Probiogenomics, Department of Chemistry, Life Sciences and Environmental Sustainability, University of Parma, 43124 Parma, Italy; gabriele.lugli@genprobio.com (G.A.L.); marco.ventura@unipr.it (M.V.)

**Keywords:** bacteriophage, *Lactococcus lactis*, dairy fermentation, cheese, infection

## Abstract

Dairy fermentations constitute a perfect “breeding ground” for bacteriophages infecting starter cultures, particularly strains of *Lactococcus lactis.* In modern fermentations, these phages typically belong to one of three groups, i.e., the 936, P335, and c2 phage groups. Traditional production methods present fewer chemical and physical barriers to phage proliferation compared to modern production systems, while the starter cultures used are typically complex, variable, and undefined. In the current study, a variety of cheese whey, animal-derived rennet, and vat swab samples from artisanal cheeses produced in Sicily were analysed for the presence of lactococcal phages to assess phage diversity in such environments. The complete genomes of 18 representative phage isolates were sequenced, allowing the identification of 10 lactococcal 949 group phages, six P087 group phages, and two members of the 936 group phages. The genetic diversity of these isolates was examined using phylogenetic analysis as well as a focused analysis of the receptor binding proteins, which dictate specific interactions with the host-encoded receptor. Thermal treatments at 63 °C and 83 °C indicate that the 949 phages are particularly sensitive to thermal treatments, followed by the P087 and 936 isolates, which were shown to be much less sensitive to such treatments. This difference may explain the relatively low frequency of isolation of the so-called “rare” 949 and P087 group phages in modern fermentations.

## 1. Introduction

In modern dairy fermentation facilities, plant design, bacterial starter cultures, sanitation regime, equipment and materials are all carefully chosen, monitored and controlled in order to limit production inconsistencies and achieve optimal product output. In such processes, the incoming raw milk is often subjected to thermal treatments, such as pasteurisation to reduce the microbial load followed by the deliberate introduction of selected starter bacteria or mixed cultures which acidify the milk and impart desirable organoleptic attributes upon the product. In contrast to this, traditional and artisan cheese manufacturing processes employ a diverse array of conditions in order to adhere to traditional production practices and/or to acquire “protected designation of origin” (PDO) status. For example, in some cases fermentations may rely solely on the acidifying activity of the autochthonous microbiota of the fermentation environment or wooden vessels for the production of cheeses with particular organoleptic and physical properties. In other cases, defined cultures and/or stainless steel vats may be employed while still retaining certain aspects of the traditional fermentation practices to achieve typical flavours and/or textures [1]. The variety of conditions under which such artisan cheeses are produced creates opportunities for phage proliferation. Furthermore, the production of certain traditional and regional artisan cheeses involves the application of raw (i.e., unpasteurized) milk and animal-derived rennet to increase the rate of coagulation of the curd (as opposed to industrially produced, recombinant enzymes). The traditional Sicilian cheeses PDO Vastedda della Valle del Belìce (referred to herein as Vastedda), Canestrato Siciliano (Canestrato), PDO Pecorino Siciliano (Pecorino) and Caciocavallo Palermitano (Caciocavallo) may be classified among such artisanal cheeses as they are regional cheeses that employ raw sheep, goat and cow’s milk in their production and often incorporate animal-derived rennets. Vastedda and Caciocavallo are stretched cheeses, while Pecorino and Canestrato cheeses are pressed cheeses and Pecorino additionally undergoes a cooking step after moulding. Additionally, wooden vats are traditionally used in the production of these cheeses, a practice that further contributes to the microbiota of the fermentation [1,2,3]. Beyond the biological hurdles presented in modern production facilities and procedures, the only major limitation to phage proliferation in traditional practices is the availability of a suitable host.

Lactococcal phages are currently classified into ten taxonomic groups based on morphology and nucleotide sequence relatedness [4]. Among these, several phage isolation studies have reported that members of the 936, c2 and P335 groups are the most frequently encountered in the dairy fermentation environment [5,6,7,8,9]. In contrast, members of the remaining seven lactococcal phage groups are much less frequently encountered [4,6,10,11]. The description of a lactococcal phage with a distinctively long tail in 1951 [12] marked the first observation of what is now called the 949 group of lactococcal phages. This isolate was shown to exhibit a tail with an estimated length of 560–610 nm, which far exceeds the typical tail length of lactococcal *Siphoviridae* phages, which normally ranges in the region of 150–200 nm [4]. More than 60 years later, the first genome sequence of a member of this group was published [13], revealing a genome of 114,768 kb with limited homology to other lactococcal phages. Subsequently, two additional members of this group have been genomically characterised, namely phiL47 and WRP3 [11,14], which were isolated from grass and a Sicilian cheese whey, respectively. The latent period of 949 (period during which phages replicate and assemble inside the host cell), was calculated to be approximately 70 min, which may in part explain the irregularity of its apparent occurrence [8]. The P087 group has currently only one described member, its namesake P087 [15,16], representing a *Siphoviridae* phage with a broad appendage at its distal tail end, called a baseplate, which is reminiscent of those of the sub-group II P335 phages Tuc2009 and TP901-1 [17,18,19,20,21]. The genomes of five members of the 1706 group of lactococcal phages have been sequenced recently and significant diversity is observed between the groups namesake 1706 and the more recently sequenced members (P118, P078, P092 and P162) bearing at most 49% nucleotide homology [22,23]. These 1706 phage group members were isolated from raw milk (P118, P078, P092 and P162) and cheese (1706). For the remaining four rare lactococcal phage groups (P034, Q54, KSY1 and 1358), only one representative genome sequence is available, impeding in-depth analysis of the diversity of these phage groups [10,24,25,26].

The current study reports on the isolation of a variety of lactococcal phages from the 936, P087 and 949 groups from cheese whey and rennet samples associated with the production of artisanal cheeses in Sicily. The genomes of two 936 as well as six P087 and ten 949 group phages were sequenced and compared to previously reported members of their respective groups. This study highlights the significance of hurdle technology in reducing microbial loads in the factory environment and the observed sensitivity of the rare phages to thermal treatments such as pasteurisation. Furthermore, the potential sources of phages in the specific artisanal production environment are discussed.

## 2. Materials and Methods

### 2.1. Bacteria and Phages

Lactococcal host strains were grown without agitation at 30 °C in M17 broth (Oxoid Ltd., Hampshire, UK) supplemented with 0.5% glucose (GM17).

Phages were propagated on the appropriate *Lactococcus lactis* indicator strains, which had been grown to an approximate optical density (OD_600 nm_) of 0.15 in 10 mL GM17 broth. Calcium chloride was added to a final concentration of 10 mM prior to infection of the culture with approximately 10^7^ plaque forming units (pfu) of the relevant phage and was incubated at 30 °C or room temperature until lysis had occurred. The lysates were filtered through a 0.45 μm filter to remove any residual bacterial debris and stored at 4 °C.

Plaque assays were performed using the previously described double agar method [27]. This method was also applied for host range analysis performed against a collection of 25 *L. lactis* strains (only sensitive strains listed in Table 1).

### 2.2. Phage Screening

A series of 12 samples of cheese whey derived from 11 Sicilian cheese production facilities and 11 samples of rennet from lamb and kid sources were collected in 2014. Similarly, seventeen whey samples and ten rennet samples sourced from the same factories as described above were collected in 2016. The whey samples associated with the production of Vastedda, Caciocavallo and Pecorino were derived from fermentations performed in wooden vats while the whey samples from Canestrato cheese were derived from stainless steel vats. Each of the 50 samples was tested against a panel of 25 *L. lactis* strains to isolate as wide a diversity of lactococcal phages as possible. The bacterial strains selected for this analysis were of known genotypes based on cell wall polysaccharide (CWPS) operon sequences from a previous study, as these are known to encode the receptor material for a variety of lactococcal phages [11,29]. Phage screening was performed using the standard double agar plaque assay as previously described [27] and a number of single plaque isolates from phage-containing samples were propagated on the relevant lactococcal host and plaque-purified at least twice to ensure homogeneity of the phage population. Of the 25 strains applied in the initial screening, only four strains were susceptible to phages from the samples (Table 1) and these were used as the propagating hosts. Phage titres of at least 10^7^ pfu·mL^−1^ were considered acceptable for application in subsequent host range analysis. In this process, the ability of the individual phage isolates to infect a panel of 25 lactococcal strains was achieved using 10 µL drops of the phage lysates in spot assays as described previously [30]. Potential phage-positive samples were confirmed by plaque assays as described above using the relevant host bacterial strain(s). Isolated phages and their host strains are presented in Table 1.

### 2.3. Selection of Phage Isolates for Genome Sequencing

A previously described multiplex polymerase chain reaction (PCR) system [31] was employed to define if the phages isolated in this study belonged to one of the three major groups of lactococcal phages, i.e., the 936, c2 or P335 groups and employing suitable positive controls for the reaction (sk1 as a representative of the 936 phage group; c2 as a representative of the c2 phage group; and Tuc2009 as a representative of the P335 phage group). Where multiple isolates from the same sample were identified as one of the three major groups, representatives were randomly selected for genome sequence analysis. Isolates that did not yield an amplicon were considered potentially novel and representatives of isolates from the phage-positive samples were selected based on possessing distinct (although in some cases overlapping) host ranges and/or restriction profiles using EcoRV, EcoRI, and HindIII as per manufacturer’s instructions (Roche, Mannheim, Germany).

### 2.4. DNA Preparation and Genome Sequencing

DNA for sequencing of the selected phage genomes was extracted from 20 mL of fresh phage lysate (>10^8^ pfu·mL^−1^) as described previously [11]. For genome sequencing, five µg of extracted DNA was used as verified by nanodrop quantification. Confirmatory molecular ID tests were also conducted on the DNA extract prior to shipment to the sequencing facility (GenProbio, Parma, Italy). The MIRA (mimicking intelligent read assembly) software program (version 4.0.2) [32] was used for de novo assembly of MiSeq-derived phage genome sequences to generate a consensus sequence. Quality improvement of the genome sequences involved customized Sanger sequencing (Eurofins, Ebersberg, Germany) of PCR products across the genomes of the phages to ensure correct assembly, double stranding and the resolution of any remaining base-conflicts occurring within homopolymeric tracts. Protein-encoding open reading frames (ORFs) were predicted using a combination of the methods Prodigal v2.6 [33] and BLASTX [34] followed by manual assessment, curation, and correction of the predicted open reading frames. A functional annotation of ORFs was performed on the basis of BLASTP [35] analysis against the non-redundant protein database (nr) provided by the National Centre for Biotechnology Information (located at: http://blast.ncbi.nlm.nih.gov/Blast.cgi) as well as using the MEGAnnotator pipeline [36]. The proposed functions of many ORFs were further validated by querying protein domain databases Pfam [37], the National Center for Biotechnology Information (NCBI) Conserved Domain Database [38], and by performing homology prediction searches using HHPred [39]. The genomes were scanned for the presence of potential transfer RNA (tRNA) genes using tRNA scan SE [40]. The genomic characteristics of the sequenced phage isolates are presented in Table 2.

### 2.5. Genbank Accession Numbers

Genbank accession numbers for the sequenced phage genomes are presented in Table 2.

The genomes of phages were compared using nucleotide BLAST analysis of the entire genomes, and comparison of the encoded individual proteins was performed by all-against-all, bi-directional BLAST alignment [41] with an alignment (or *E*-value) cut-off value of 0.0001 and greater than 50% identity across at least 50% of the amino acid sequence. Multiple alignment of nucleotide sequences of the 949 and P087 phages isolated in this study and those of previously sequenced members of these groups (949 [13], L47 [14], WRP3 [11] and P087 [16]) were performed using ClustalW software. The alignment was employed to generate an unrooted phylogenetic tree using the “itol” software (http://itol.embl.de/), applying the neighbor-joining method. The Markov Clustering (MCL) algorithm was executed via the mclblastline pipeline v12-0678 as described previously applying a cut-off value of 50% amino acid identity across 50% of the protein [42]. A binary matrix containing presence/absence of each MCL family in all sequenced phages belonging to the 949 and P087 groups was first computed and used as input of MeV suite (V4.9) [43]. The produced MCL matrix was visualized using TMev4 and analysed using a two-way hierarchical clustering (HCL) to group phage genomes according to their similarity at the protein level. Pan/core genome analysis was performed using PGAP v1.0 [44] according to Heaps law pan-genome model [45]. The ORF content of each genome was organised in functional gene clusters via the Gene Family method. ORFs, which produce an alignment with at least 50% sequence identity across 50% of the gene/protein length were clustered and a pan/core genome profile was generated.

### 2.6. Thermal Inactivation Assays

The ability of selected phages isolated in this study to withstand thermal processing treatments that are widely employed in the dairy industry was assessed as follows. 0.1 mL of a fresh phage preparation (10^7^–10^8^ pfu·mL^−1^ propagated the day before the assays were performed) was suspended in 0.9 mL of preheated 10% reconstituted skimmed milk (RSM) and exposed to three sets of conditions: (i) room temperature for 30 min (untreated control); (ii) 63 °C × 30 min (low temperature long time, LTLT, pasteurization temperatures) or; (iii) 83 °C × 10 min (treatment used in yoghurt manufacture). The phage titre following exposure to the thermal treatments was determined by plaque assay as described above and compared to the untreated control, which was held at room temperature for 30 min. Three independent assays were performed for each phage analysed in this study.

## 3. Results

### 3.1. Phage Survey

To assess the potential sources of phages within the production process, swabs from the wooden fermentation vats, whey samples, and animal-derived rennet samples were tested for the presence of lactococcal phages. Raw milk was not tested as such samples were not available, although it would be expected that the raw milk would contribute to the biodiversity of phages in a given fermentation facility [22]. To compensate for this, whey samples derived from cheese factories using raw cow’s milk (Canestrato and Caciocavallo cheeses), raw ewe’s milk (Vastedda and Pecorino cheeses), and raw goat’s milk, were tested to explore the potential diversity between the factories based on the raw milk type used in the production process. As expected (and described above), the majority of phages were isolated from the whey samples where the appropriate propagation host(s), medium and conditions are provided. Thus, fifty samples of cheese whey and rennet employed in traditional cheese-making processes in Sicily in 2014 and 2016 were assessed for the presence of phages against a panel of 25 lactococcal strains. Through this analysis, 59 phage isolates (27 from the 2014 samples and 32 from the 2016 samples) were recovered and propagated on the relevant sensitive host. Of the 22 whey samples assessed, 15 (68%) were phage positive against at least one lactococcal strain from the collection (Table 3). Similarly, four of the 28 rennet samples assessed (14%) contained phages capable of infecting at least one lactococcal host strain from the collection. Details of the samples from which phages were isolated in this study are presented in Table 1, while the titres of phages recovered from the phage-positive samples are presented in Table 3.

### 3.2. Diversity Assessment of the Phage Collection

In total, 59 individual plaque isolates were propagated on their sensitive hosts and those that were capable of producing a high titre (at least 10^7^ pfu·mL^−1^) were applied to a host range analysis against the previously mentioned panel of lactococcal strains. In addition, characterisation by DNA restriction profiling and multiplex PCR typing were also performed to ascertain the genetic diversity among the isolated phage collection. These profiling strategies resulted in the identification of eight 936 phage isolates and 51 phages that did not generate an amplicon according to the multiplex PCR system (data not shown). Host range analysis using a panel of 25 lactococcal strains was undertaken to determine if different sensitivity profiles could be established and used as a differentiating tool. Based on distinctive restriction profiles and/or host ranges, 18 phage isolates were selected for genome sequence analysis. Among these were two representative 936 phage isolates and 16 phage isolates of unknown phage group(s) with distinct (or overlapping) host ranges and genome restriction profiles. The host ranges of the phages selected for genome sequencing are presented in Table 4 (with sensitive strains only presented).

The genome sequences of the new isolates were first compared to those available in public databases through BLASTN analysis. This revealed that among the 16 sequenced phages that could not be typed according to multiplex PCR system available for the three main groups of lactococcal phages (i.e., P335, 936 and c2), six belong to the P087 group and ten to the 949 group of lactococcal phages (thus explaining their failure to generate an amplicon employing this multiplex PCR approach). The genome characteristics of the individual phages are presented in Table 2. To assess if a link exists between the dominant phage groups isolated from various whey samples and the raw milk used in its production, we examined the groups of phages isolated from the different cheese wheys. 949 group phages were isolated from Canestrato (raw cow’s milk), Vastedda (raw ewe’s milk) and Caciocavallo (raw cow’s milk) cheese wheys indicating their widespread presence in all cheese types irrespective of the source of the milk or factory site tested. Similarly, the P087 isolates were found in Vastedda and Caciocavallo cheese whey samples highlighting their ubiquity in artisanal cheese production in Sicily. Swabs of the wooden vats did not contain phages capable of infecting the strains in our collection although the relatively low number of samples taken and the selection of test strains applied may have additionally influenced the results. Interestingly, four rennet samples were found to be positive for phages with typical titres of 10^2^–10^3^ pfu·mL^−1^ observed on the lactococcal strain SMQ-86. Rennet samples derived from lamb (ten samples) and kid (one sample) were tested and all positive samples were isolated from the lamb rennets (likely a reflection of the larger proportion of lamb rennet samples tested). Interestingly, the eight individual phages isolated from lamb rennets were all confirmed as 936 group phages and were the only 936 group phages encountered in this study. Therefore, it is clear that the raw materials employed in traditional cheese production systems in Sicily provide a diverse range of lactococcal phage groups including those that are generally regarded as rare.

**Figure 1:**
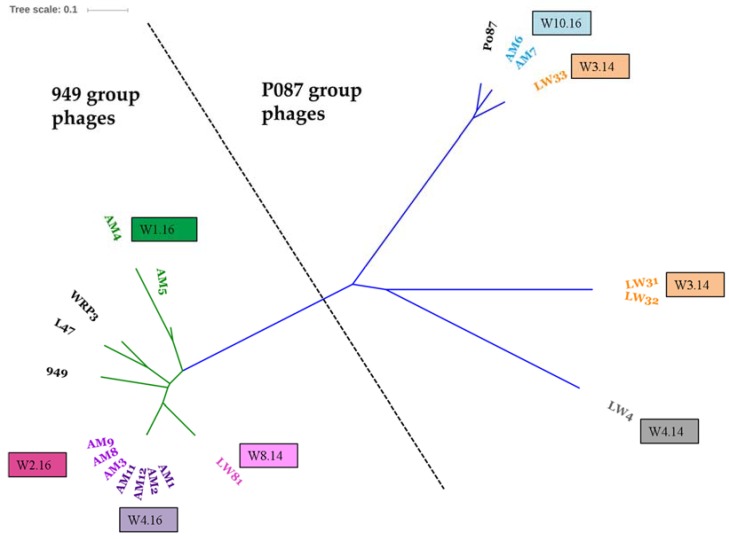
Unrooted phylogenetic tree of the complete nucleotide sequences of the 949 (green branches) and P087 (blue branches) isolates from this study and previously sequenced members 949, L47, WRP3, and P087. Whole phage nucleotide alignment was performed via ClustalW V2.1. Subsequently, the phylogenetic tree was generated using the neighbor-joining method and bootstrapped (×1000) replicates. Visualisation of the phylogenetic tree was performed using the Itol software (http://itol.embl.de/). Clusters of phage genomes by source sample are indicated in coloured text and corresponding coloured text boxes with the sample from which the phages were derived. The 949 phages isolated from W1.16 (green) are genetically distinct from those isolated from samples W2.16 (hot pink), W4.16 (purple) and W8.14 (pale pink). Similarly, the P087 phage isolates are genetically distinct based on the sample source with the exception of LW33 (isolated from W3.14, orange), which groups more closely to the 2016 isolates from W10.16 (blue) and P087, while phage LW4 occupies a distinct position (grey) highlighting its more distant relationship to the other P087 isolates.

The genome (and deduced proteome) of each phage was compared to all sequenced members of its related group using BLASTN and BLASTP analysis to identify the overall relatedness to other group members at the nucleotide and protein levels. Figure 1 is an unrooted phylogenetic tree of the nucleotide sequences of 949 and P087 phage genomes arising from this study and also previously sequenced members of the two groups, namely 949, L47 and WRP3 from the 949 group, and P087, the namesake of its group. The tree highlights the genomic conservation of the phage isolates from both groups. Perhaps unsurprisingly, phages isolated from the same cheese whey sample display significant similarity and while low numbers of single nucleotide polymorphisms are observed, it is likely that the population of 949 or P087 phages within a single sample (representing a single production round) is descended from one closely related phage ancestor either from that production round or the factory environment.

**Figure 2:**
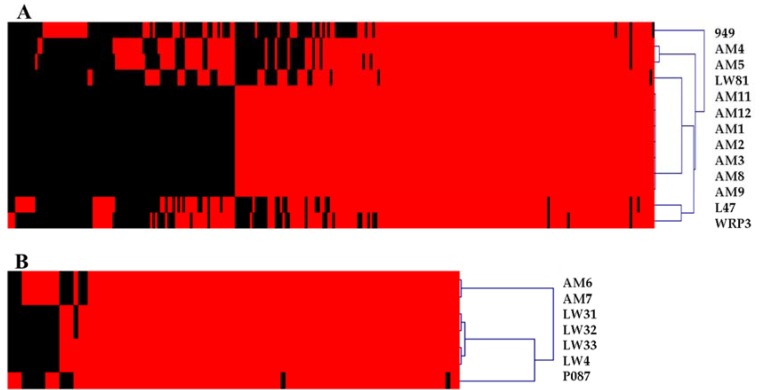
Heatmap indicating the presence (red) or absence (black) of individual protein families encoded by members of the 949 (**A**) and P087 (**B**) groups. Distinct phylogenetic clusters of the 949 and P087 phages can be identified using this approach that are consistent with the phylogeny based on overall nucleotide content presented in Figure 1.

To define the extent of the genetic diversity within the 949 and P087 phage groups that now possess thirteen and seven completely sequenced members, respectively, core/pan-genome analysis was undertaken. The core genome analysis incorporates both the predicted genes and their products to define the conserved (and dispensable) elements of the genome of a given group of organisms. Within the 949 group of phages, 94 genes were defined as the core genome (based on a cut-off of 50% amino acid identity across 50% of the gene/protein), representing at least half of the genome of 949 isolates, which encode between 160 and 195 genes (Figure S1A). Using the same approach, 74 core genes were defined for the P087 phage isolates, which constitute at least 80% of the predicted genome of the P087 phage genomes (Figure S1B). This highlights the genetic conservation of this group among the currently sequenced isolates. To assess if further (significant) genetic novelty may be expected within a group of organisms, a pan-genome analysis was performed. In the current analysis, the exponential value was calculated to be 0.21 and 0.021 for the 949 and P087 phages [45], respectively, indicating that the pan-genomes of these groups are closed (where a *x^n^* value above 0.5 is considered open), that little diversity is observed among these phage groups (Figure S2A and S2B). A phylogenetic tree of the 949 and P087 isolates was also constructed based on the presence or absence of protein families identified for these phage groups through MCL analysis and the resulting trees were consistent with the nucleotide-based phylogenetic analysis (Figure 1 and Figure 2).

### 3.3. Phage-Host Interactions of the Isolated Phages

**Figure 3:**
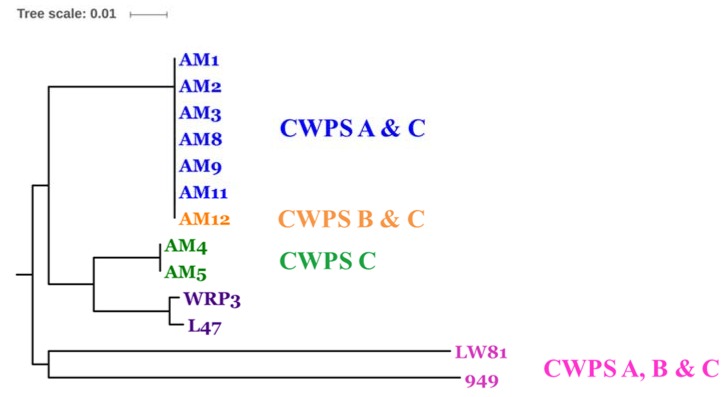
Rooted phylogenetic tree (rooted by the first sequenced member, 949) of the receptor binding proteins (RBPs) encoded by the 949 phage isolates from this and previous studies. The tree was constructed based on a multiple alignment using ClustalW software with the neighbour-joining method with a bootstrap value of 1000. Visualisation of the phylogenetic tree was performed using the ITOL software (http://itol.embl.de/). The names of the phage isolates derived from this study are colour-coded to indicate the cell wall polysaccharide (CWPS)-type presented by host(s) of these phages as indicated in the text, while in purple text are previously described isolates whose host CWPS types were not assessed in this study.

To further understand the link between the host range of the phages and their corresponding receptor binding protein (RBP), a host range survey of the isolated phages and a detailed analysis of the RBPs and their phylogeny was undertaken between phages of different taxonomic groups (Table 4 and Figure 3). The RBP-encoding gene of each of these phage groups have previously been proposed and/or experimentally proven, and is detailed below. Phylogenetic analysis of the RBPs of the identified 949 phages including those of the previously sequenced members of this group correlated well with the host range as phages AM4 and AM5, which display a distinctly narrow host range relative to most of the other 949 isolates, and also exhibit a distinct RBP phylogeny. Conversely, the 949 phages AM1, AM2, AM3, AM8, AM9, AM11 and AM12 RBPs are highly related (Figure 3) and similarly share common host strains (Table 4). However, despite the relatedness of their RBPs, there are unique elements to each of the phages’ host range which may be reflective of the presence of phage-resistance mechanisms within the host strain(s) including restriction and modification or abortive infection systems. Such systems may limit the ability of certain phage isolates to infect the host or the substitution of critical residues within the host-interacting domain of the RBPs of these phages. Finally, isolate LW81, which occupies a distinct position on the phylogenetic tree, displays a unique host range relative to the other isolates and is the only 949 isolate in this study capable of infecting lactococcal strains expressing a CWPS A, B or C type (Figure 3 and Table 4). Overall, a number of strains possessing a type C CWPS are infected by members of this group.

The RBP of P087 was identified in a previous study as gp78_P087_ and was confirmed in this study using a structural bioinformatic approach using HHPred software [39] with the amino acid sequences of all the predicted structural proteins encoded by AM6 as a representative of the group. Through this analysis, the RBP was identified as ORF80_AM6_ with 97.4% probability, as it exhibits a 3da0_A domain (which is found in the confirmed RBPs of the P335 and 936 phages, TP901-1 and p2). The RBP of P087 and the newly isolated P087 group phage isolates bear 99% amino acid homology. This was unexpected given the distinct (if overlapping) host ranges of AM6, AM7, and LW31, LW32, LW33, and LW4. The observed differences in host range are thus likely the result of phage-resistance systems encoded by the assessed host strains targeting specific phage isolates based on genomic regions aside from the RBP.

**Figure 4:**
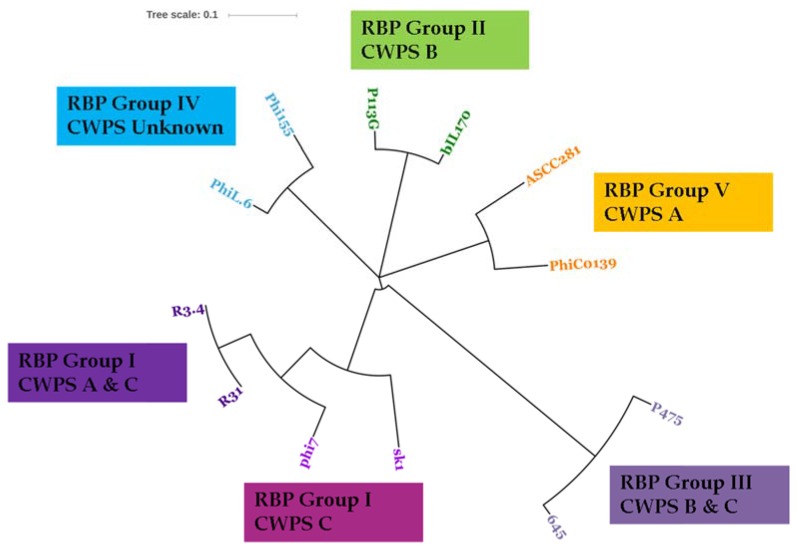
Unrooted phylogenetic tree of the RBP sequences of R31 and R3.4 and previously sequenced representative members of the five 936 RBP groups. R31 and R3.4 RBPs are identical to each other and occupy a distinct node within the RBP group I clade. The CWPS types of the host strains infected by these phages are presented along with the RBP grouping information.

The RBP of the two sequenced 936 isolates are 100% identical across the complete predicted protein sequences, which is consistent with their identical host ranges (Table 4). BLASTP analysis of the RBP of these phages identifies the RBP of the 936 phage phi7 as the closest homologue with 90% identity across the full length of the proteins (238 of 264 amino acids are identical). In a previous study, the RBP of phi7 was classified as RBP group I, which comprises a group of phages that were previously observed to display a preference for host strains with a C-type CWPS [29]. Notably, in addition to infecting a C-type CWPS strain, the two sequenced 936 phages infect *L. lactis* SMQ-86, which exhibits an A-type CWPS genotype and the ability to infect the A-type CWPS strain may be due to the specific amino acid sequence differences observed in these phage RBPs. Furthermore, a recent study expanding on previous RBP groupings of the 936 phages identified five RBP subgroups, none of which were observed to correlate with the ability to infect both A- and C-type CWPS possessing strains [46]. Therefore, it is possible that R31 and R3.4 RBPs represent a sixth group of the 936 RBP genotypes. To investigate if R31 and R3.4 RBPs represent a sixth group of the 936 RBP genotypes or a subgroup of previously identified RBP groups, the amino acid sequences of representatives of each of the previously identified five RBP subgroups were compared to those of R31 and R3.4 to determine their phylogeny. This analysis revealed that the RBPs of R31 and R3.4 cluster closely with the RBP group I phages phi7 and sk1, and are likely variants of this RBP group with an expanded host range (Figure 4).

### 3.4. Thermal Inactivation of Phages

**Figure 5:**
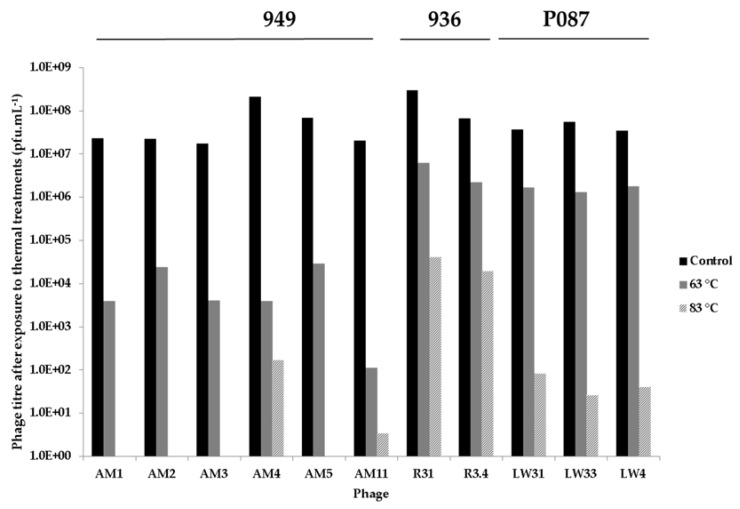
Thermal inactivation assays of selected 949, P087, and 936 isolates at room temperature (control, black bars), 63 °C (grey) and 83 °C (patterned). The 949 isolates display almost complete inactivation at 83 °C and reduced titres at 63 °C. The P087 isolates exhibit approximately 5 log titre at 83 °C and only minor titre reductions (one to two logs) at 63 °C.

In this study, it is hypothesised that the relatively low frequency with which the 949 and P087 phage groups are encountered in modern dairy fermentation facilities may be due, at least in part, to their sensitivity to pasteurisation and thermal treatments to which milk is subjected in many modern dairy fermentations. This is in stark contrast to the application of raw milk in the production of the artisanal cheeses of Sicily mentioned in this study thus explaining the success and relative dominance of the 949 phages in these traditional fermentations. To explore this theory, a selection of six 949, three P087 and two 936 phages isolated as part of this study were subjected to thermal treatments including 63 °C × 30 min and 83 °C × 10 min. With the exception of AM4, the 949 phage isolates were all completely inactivated at 83 °C, while exhibiting 4 to 6 log reductions in infectivity at 63 °C (Figure 5). The P087 isolates exhibited a significant reduction in infectious numbers upon treatment at 83 °C with approximately 6 log reductions in phage titres observed (Figure 5). In contrast, the 936 isolates R31 and R3.4 displayed greater survival capacities exhibiting one to two log reductions of infectivity at 63 °C and three to four log reductions in infectivity at 83 °C.

## 4. Discussion

In traditional cheese fermentations, an array of cheese- and region-specific practices are employed to produce cheeses with particular flavours and textures. While defined cultures may sometimes be employed in the production of artisan cheeses, in many cases the autochthonous bacteria present in the fermentation environment are responsible for the acidification and development of the derived cheese. In such situations, the microbial landscape is continuously in a state of flux as phage populations infect susceptible strains within the complex culture and resistant strains become dominant until the phage population adapts to infect the previously dominant strains in turn. Additionally, microbial biofilms established within the wooden fermentation vats and the incoming raw milk contribute to the microbial ecology of these fermentations [3,47]. As the starter cultures are undefined and complex, while also subject to change and factory-specific, it is impossible to precisely define the phage population by culture-dependent methods. An additional variable is the application of pasteurized or unpasteurized milk in the production of artisan cheeses. The current study was aimed at exploring the diversity of lactococcal phages present in a range of samples derived from the production of traditional Sicilian cheeses using a test panel of 25 lactococcal strains that have been employed in previous phage-host interaction studies to gain an insight into the overall phage population [11,29]. The samples were taken from whey and rennet used in the production of Canestrato, Caciocavallo, Pecorino and Vastedda cheeses produced in stainless steel (Canestrato only) and wooden vats (Caciocavallo, Vastedda and Pecorino). Through this process, a total of 59 individual plaque isolates were propagated and selected for further characterisation. Using established multiplex PCR approaches [31,48], eight isolates were identified as belonging to the 936 group (Table 1) while the remaining 51 could not be typed according to these methods.

To understand the genetic diversity of the isolated phages and to assign the untyped phage isolates to a lactococcal phage group, eighteen isolates were selected for genome sequencing based on sample source, unique/overlapping host range and/or restriction profile. Among these were two randomly selected 936 group phages isolated from lamb rennet used in Vastedda cheese production (Table 1), while the remaining 16 isolates were of unknown phage groups. Genome sequencing revealed that the untyped isolates comprised six P087 group isolates and ten 949 group isolates (Table 2). This finding is in complete contrast to several phage isolation studies that have been performed in recent years in modern global fermentation facilities [5,6,7], which revealed that members of the 936 and P335 phage groups dominate. The apparent relative dominance of 949 and P087 group phages in this study may be biased (i) by the strains naturally present in the starter culture in the fermentation, (ii) the raw milk starting material, (iii) by the application of natural rennets, which we have shown to contain phages, or (iv) by the laboratory strains used in the selection process; however, this remains a remarkable finding among phage isolation studies.

Since it is proposed that the so-called rare lactococcal phages are more often associated with raw milk and that longer tailed phages such as those represented by members of the 949 and P087 groups are susceptible to physical damage [4], it may thus be expected that these phages may be sensitive to thermal treatments that are employed in dairy fermentation processes. To examine this hypothesis, representative 936, P087 and 949 phage isolates from this study were subjected to thermal treatments. The ability of the phages to resist treatments of 63 °C × 30 min and 83 °C × 10 min was assessed and while the 936 isolates (R31 and R3.4) were relatively stable when treated at 63 °C and exhibited approximately 3-log reduction in infectivity at 83 °C, the 949 isolates showed complete loss of infectivity at the higher temperature in most cases while the P087 isolates exhibit approximately 5-log reductions in titres. The increased thermal sensitivity of these phages may be at least part of the reason that these phage groups are not regularly encountered in modern fermentation facilities where thermal processes are frequently employed.

In this study, phages were isolated from the animal derived rennet samples. This, to our knowledge, is the first description of phages isolated from rennet and it appears to act as an additional reservoir of phages in traditional processes where animal rennets as opposed to purified, recombinant enzymes of microbial origin are employed to expedite the milk coagulation and separation processes. The finding of 936 type phages in the rennet samples also serves to highlight the ability of (some) phages to withstand the relatively low pH (3.5–4) of the abomasum of the young ruminants from which the rennet is extracted. While 936 phage isolates were isolated from the rennet samples in this study, it is likely that other lactococcal phage groups also reside there but were not detected in this study, possibly due to (i) the strain selection applied, (ii) low phage numbers that may require pre-enrichment in order to be detected or (iii) a progressive inactivation due to prolonged exposure to low pH.

From this study, it is clear that the traditional and artisanal cheese production processes permit the proliferation and even an apparent dominance of so-called rare lactococcal phage groups. However, the phages isolated in this study undoubtedly represent only a proportion of the total phage population based on the collection of strains available at the time of processing. The strains that may be present within these “wild” fermentations may be considerably more diverse. Therefore, it is clear that while culture-dependent methods of analyzing such phage populations may provide a “snapshot” of the diversity of phages within such processes, culture-independent methods such as metagenomic analyses would be a useful complement to such studies.

Of the 25 lactococcal strains employed in this study, a small number are particularly sensitive to a number of phages in the collection, such as *L. lactis* 3107 and SMQ-86 (Table 4). The sequenced 936 group isolates, R31 and R3.4 infect both of these strains. The RBPs of these phages, which dictate host recognition and binding, bear most significant similarity to those of the RBP subgroup I phages, phi7 and JM2 [29]. In our previous study, phages exhibiting RBPs of subgroup I were capable of infecting host strains with a CWPS C type only, while in this study both phages infect *L. lactis* 3107 and SMQ-86, which possess CWPS C and A types, respectively [29]. This finding highlights the adaptability of the 936 phages to the evolving landscape of available hosts in its environment. Consistent with previous studies of 949 phages, the 949 isolates in this study displayed a relatively broad host range with typically more than five strains infected. The only exceptions to this general observation were phages AM4, AM5 and AM12. However, the panel of strains in this study may not be reflective of their natural host range thus reducing the apparent host range of these isolates. It is noteworthy that the 949 phages infect more CWPS C type strains than those of CWPS A or B types while the P087 isolates infect several strains of CWPS A and B types (Table 4). These findings indicate that the fermentation environment, from which the phages were derived, contain a wide diversity of lactococcal strains (expressing a variety of CWPS types) and that the phage groups dominantly isolated from particular factories may provide an insight into which lactococcal CWPS type strains are dominant at a given time.

In summary, the artisanal cheese production process incorporates several points at which phages may be introduced, propagated, and distributed to the next production cycle. The application of raw milk, animal-derived rennets, minimal processing combined with the use of wooden vats and back-slopping (if used) introduce the possibility of increasingly complex microbial populations. This is reflected in the identification of a significant collection of 949 and P087 phage isolates in whey samples derived from Sicilian artisanal cheese production facilities in the present study. The heat labile nature of the 949 and P087 isolates in this study relative to the 936 isolates serves to further highlight the adaptability of the 936 phages to modern fermentation facilities while simultaneously explaining the “rarity” of the 949 and P087 phages.

## Figures and Tables

**Table 1:** Phages isolated in this study.

Phage	*Lactococcus lactis* Host (Reference)	Source (Sample Ref. No.)	Year
LW11	3107 [15]	Vastedda cheese whey (raw ewe’s milk) (W1.14)	2014
LW12	3107	Vastedda cheese whey (W1.14)	2014
LW21 *	3107	Vastedda cheese whey (W2.14)	2014
LW22*	3107	Vastedda cheese whey (W2.14)	2014
**LW31**	3107	Vastedda cheese whey (W3.14)	2014
**LW32**	3107	Vastedda cheese whey (W3.14)	2014
**LW33**	3107	Vastedda cheese whey (W3.14)	2014
LW34	3107	Vastedda cheese whey (W3.14)	2014
**LW4**	3107	Canestrato cheese whey (raw cow’s milk) (W4.14)	2014
LW71 *	3107	Caciocavallo cheese whey (raw cow’s milk) (W7.14)	2014
LW72 *	3107	Caciocavallo cheese whey (W7.14)	2014
**LW81**	3107	Canestrato cheese whey (W8.14)	2014
LW82	3107	Canestrato cheese whey (W8.14)	2014
LW91	3107	Canestrato cheese whey (W9.14)	2014
LW92 *	3107	Canestrato cheese whey (W9.14)	2014
LW101	3107	Canestrato cheese whey (W10.14)	2014
LW102	3107	Canestrato cheese whey (W10.14)	2014
GW11 *	3107	Goat’s cheese whey (W11.14)	2014
CW12 *	3107	Cow’s cheese whey (W12.14)	2014
R1	SMQ-86 [4]	Lamb rennet used in Caciocavallo cheese (R1.14)	2014
R2	SMQ-86	Lamb rennet used in Caciocavallo cheese (R2.14)	2014
**R31**	SMQ-86	Lamb rennet used in Vastedda cheese (R3.14)	2014
R32	SMQ-86	Lamb rennet used in Vastedda cheese (R3.14)	2014
R33	SMQ-86	Lamb rennet used in Vastedda cheese (R3.14)	2014
**R3.4**	SMQ-86	Lamb rennet used in Vastedda cheese (R3.14)	2014
R35	SMQ-86	Lamb rennet used in Vastedda cheese (R3.14)	2014
R4	SMQ-86	Lamb rennet used in Caciocavallo cheese (R4.14)	2014
**AM1**	3107	Vastedda cheese whey (W4.16)	2016
**AM2**	3107	Vastedda cheese whey (W4.16)	2016
**AM3**	3107	Caciocavallo cheese whey (W2.16)	2016
**AM4**	3107	Caciocavallo cheese whey (W1.16)	2016
**AM5**	3107	Caciocavallo cheese whey (W1.16)	2016
**AM6**	SMQ-86	Caciocavallo cheese whey (W10.16)	2016
**AM7**	SMQ-86	Caciocavallo cheese whey (W10.16)	2016
**AM8**	SMQ-86	Caciocavallo cheese whey (W2.16)	2016
**AM9**	SMQ-86	Caciocavallo cheese whey (W2.16)	2016
AM10	3107	Caciocavallo cheese whey (W2.16)	2016
**AM11**	3107	Caciocavallo cheese whey (W2.16)	2016
**AM12**	IL1403 [28]	Caciocavallo cheese whey (W2.16)	2016
AM13	C10 [29]	Vastedda cheese whey (W4.16)	2016
AM14	C10	Caciocavallo cheese whey (W5.16)	2016
AM15	C10	Caciocavallo cheese whey (W5.16)	2016
AM16	C10	Caciocavallo cheese whey (W5.16)	2016
AM17	C10	Caciocavallo cheese whey (W5.16)	2016
AM18	C10	Caciocavallo cheese whey (W5.16)	2016
AM19	SMQ-86	Caciocavallo cheese whey (W2.16)	2016
AM20	SMQ-86	Caciocavallo cheese whey (W2.16)	2016
AM21	SMQ-86	Caciocavallo cheese whey (W10.16)	2016
AM22	SMQ-86	Caciocavallo cheese whey (W10.16)	2016
AM23	SMQ-86	Caciocavallo cheese whey (W10.16)	2016
AM24	3107	Caciocavallo cheese whey (W1.16)	2016
AM25	3107	Caciocavallo cheese whey (W1.16)	2016
AM26	3107	Caciocavallo cheese whey (W1.16)	2016
AM27	3107	Caciocavallo cheese whey (W1.16)	2016
AM28	3107	Caciocavallo cheese whey(W1.16)	2016
AM29	3107	Caciocavallo cheese whey (W2.16)	2016
AM30	3107	Vastedda cheese whey (W4.16)	2016
AM31	3107	Vastedda cheese whey (W4.16)	2016
AM32	3107	Vastedda cheese whey (W4.16)	2016

* Denotes phage isolates that produced low titres (10^6^ plaque forming units (pfu)·mL^−1^ or lower) and were deemed unsuitable for application in subsequent host range analysis. Phage isolate names highlighted in bold-face text are those that were selected for genome sequencing.

**Table 2:** Genomic characteristics of sequenced phages in this study.

Phage	Phage Group	Genome Length (kb)	GC %	No. Predicted ORFs	tRNAs	Best Hit (% nt ID/Coverage)	Genbank Accession No.
**LW31**	P087	60.551	34.3	85	3 (Cys, Asn, Thr)	P087 (97/91)	KY554762
**LW32**	P087	60.161	34.3	87	3 (Cys, Asn, Thr)	P087 (97/91)	KY554763
**LW33**	P087	59.899	34.4	86	3 (Cys, Asn, Thr)	P087 (97/91)	KY554764
**LW4**	P087	60.217	34.3	88	3 (Cys, Asn, Thr)	P087 (97/91)	KY554765
**LW81**	949	128.179	32.6	178	4 (Trp, Asp)	WRP3 (95/84)	KY554777
**R31**	936	27.203	35.4	52	2 (Trp, Pro)	Phi19 (90/82)	KY554761
**R3.4**	936	27.704	35.4	51	2 (Trp, Pro)	Phi19 (96/82)	KY554760
**AM1**	949	125.658	32.6	178	3 (Pro, Trp)	WRP3 (95/82)	KY554768
**AM2**	949	125.656	32.5	177	3 (Pro, Trp)	WRP3 (95/82)	KY554769
**AM3**	949	126.032	32.5	177	3 (Pro, Trp)	WRP3 (95/82)	KY554770
**AM4**	949	132.949	32.5	194	3 (Arg, Pro, Met)	WRP3 (94/81)	KY554771
**AM5**	949	128.178	32.6	182	3 (Arg, Pro, Met)	WRP3 (94/81)	KY554772
**AM6**	P087	62.054	34.3	90	4 (Pro, Thr, Asn, Cys)	P087 (97/96)	KY554766
**AM7**	P087	62.252	34.2	90	4 (Pro, Thr, Asn, Cys)	P087 (97/96)	KY554767
**AM8**	949	126.177	32.6	178	3 (Pro, Trp)	WRP3 (95/82)	KY554773
**AM9**	949	125.294	32.5	179	3 (Pro, Trp)	WRP3 (95/82)	KY554774
**AM11**	949	126.161	32.6	178	3 (Pro, Trp)	WRP3 (95/82)	KY554775
**AM12**	949	125.842	32.6	179	3 (Pro, Trp)	WRP3 (96/82)	KY554776

ORFs: open reading frames; tRNAs: transfer RNAs.2.6. Comparative Genomic Analysis.

**Table 3:** Phage titres on strains that were sensitive to phages and associated plaque morphologies.

Sample	Phage Titre on Lactococcal Strain (pfu·mL^−1^)				Plaque Morphology
	3107	SMQ-86	C10	IL1403	
W1.14	6 × 10^3^	-	-	-	1 mm clear
W2.14	8 × 10^2^	-	-	-	Pinpoint–0.5 mm
W3.14	4 × 10^2^	2 × 10^3^	-	-	1 mm
W4.14	1 × 10^2^	-	-	-	1 mm
W7.14	1.3 × 10^4^	-	-	-	Pinpoint–1 mm
W8.14	4 × 10^3^	-	-	-	Pinpoint–1 mm
W9.14	2 × 10^3^	-	-	-	Pinpoint and 1.5 mm
W10.14	2.3 × 10^3^	-	-	-	Pinpoint and 1 mm
W11.14	-	8 × 10^2^	-	-	Pinpoint
W12.14	-	2.7 × 10^3^	-	-	Pinpoint
R1.14	-	3.3 × 10^3^	-	-	1.5 mm
R2.14	-	5.9 × 10^3^	-	-	1.5 mm
R3.14	-	1.4 × 10^3^	-	-	1.5 mm
R4.14	-	7 × 10^2^	-	-	1.5 mm
W1.16	8 × 10^2^	-	-	-	Pinpoint
W2.16	4 × 10^2^	4 × 10^2^	-	1 × 10^2^	Pinpoint
W4.16	5 × 10^2^	-	1 × 10^2^	-	Pinpoint
W5.16	-	-	5 × 10^2^	-	Pinpoint
W10.16	-	5 × 10^2^	-	-	1 mm

**Table 4:** Host range of the sequenced phages.

Phage	Phage Titre on Lactococcal Strains (pfu·mL^−1^)															
	CWPS A Strains				CWPS B Strains			CWPS C Strains								
	SMQ-86	SMQ-562	WM1	275	UC77	IL1403	223	3107	KH	NZ9000	R1k10	W34	FD13	JM3	1196	158
**R31 ^a^**	**4 × 10^8^**							6 × 10^8^								
**R3.4 ^a^**	**3 × 10^7^**							2 × 10^8^								
**LW31 ^b^**	2 × 10^7^	3 × 10^6^			2 × 10^2^			**4 × 10^7^**								
**LW32 ^b^**	2 × 10^7^	4 × 10^6^			1 × 10^2^	4 × 10^4^		**3 × 10^7^**								
**LW33 ^b^**	4 × 10^7^	3 × 10^6^			1 × 10^2^	3 × 10^4^		**4 × 10^7^**								
**LW4 ^b^**	3 × 10^7^	2 × 10^6^			3 × 10^2^	7 × 10^3^		**5 × 10^7^**								
**AM6 ^b^**	**4 × 10^7^**															
**AM7 ^b^**	**2 × 10^6^**								2 × 10^3^							
**LW81 ^c^**	4 × 10^7^	5 × 10^5^				3 × 10^6^	4 × 10^6^	6 × 10^8^			2 × 10^5^					
**AM1 ^c^**	8 × 10^5^		2 × 10^5^					**4 × 10^7^**	1 × 10^4^	1 × 10^3^		2 × 10^3^				
**AM2 ^c^**	7 × 10^5^		7 × 10^4^					**8 × 10^7^**				5 × 10^3^	1 × 10^5^			
**AM3 ^c^**	3 × 10^5^		9 × 10^4^					**5 × 10^7^**	3 × 10^5^			1 × 10^3^	1 × 10^4^			8 × 10^2^
**AM4 ^c^**								**2 × 10^7^**								
**AM5 ^c^**								**3 × 10^7^**				3 × 10^4^				
**AM8 ^c^**	**5 × 10^7^**		3 × 10^5^	1 × 10^3^				4 × 10^6^	1 × 10^5^			4 × 10^4^	9 × 10^4^			
**AM9 ^c^**	**9 × 10^7^**		8 × 10^5^					1 × 10^7^	2 × 10^6^	1 × 10^3^		1 × 10^3^	2 × 10^5^	1 × 10^5^	7 × 10^2^	9 × 10^4^
**AM11 ^c^**			3 × 10^5^					**7 × 10^7^**				5 × 10^4^				
**AM12 ^c^**						**5 × 10^5^**		9 × 10^7^								

^a^ Indicates 936 group phages; ^b^ Indicates P087 group phages; ^c^ Indicates 949 group phages. Bold-face text is used to indicate the primary host strain on which the phage isolates were propagated.

## Supplementary Materials

The following are available online at www.mdpi.com/1999-4915/9/3/45/s1, Figure S1: Core genome analysis of the sequenced members of the 949 (Panel A) and P087 (Panel B) phage groups using the PGAP pipeline where the number of genes is plotted as a function of the number of genomes (7 P087 phage genomes and 13 949 phage genomes) sequentially added, Figure S2: Pan-genome analysis of the sequenced members of the 949 (Panel A) and P087 (Panel B) phage groups using the PGAP pipeline.
