# Supplementary material for: Phage Biodiversity in Artisanal Cheese Wheys Reflects the Complexity of the Fermentation Process
Source: Viruses. 2017 Mar 16;9(3):45. doi: 10.3390/v9030045 (PMC5371800; doi:10.3390/v9030045)
Supplement: Supplementary file 1 [file viruses-09-00045-s001.pdf]

Supplementary Materials: Phage Biodiversity in Artisanal Cheese Wheys Reflects the Complexity of the Fermentation Process

Jennifer Mahony, Angelo Moscarelli, Philip Kelleher, Gabriele A. Lugli, Marco Ventura, Luca Settanni and Douwe van Sinderen

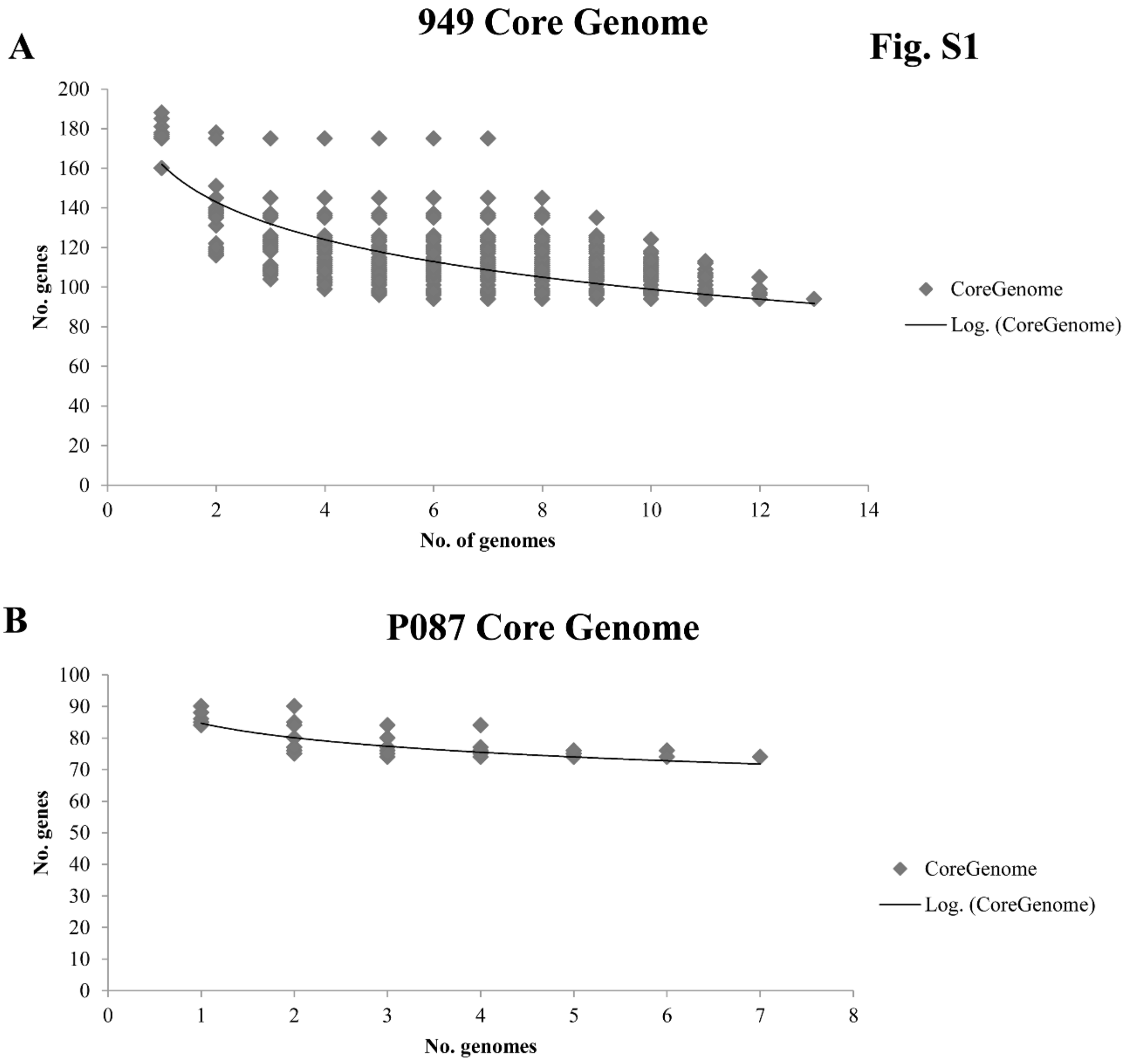

Figure S1. Core genome analysis of the sequenced members of the 949 (**A**) and P087 (**B**) phage groups using the PGAP pipeline where the number of genes is plotted as a function of the number of genomes (7 P087 phage genomes and 13 949 phage genomes) sequentially added. The number of core genes for the phage groups were determined as 94 and 74 for the 949 and P087 phage groups, respectively. The depicted curves represent the lines of best fit with R^2 values of 0.986 and 0.998 for the 949 and P087 phages, respectively. No.: Number.

Fig. S2

A

B

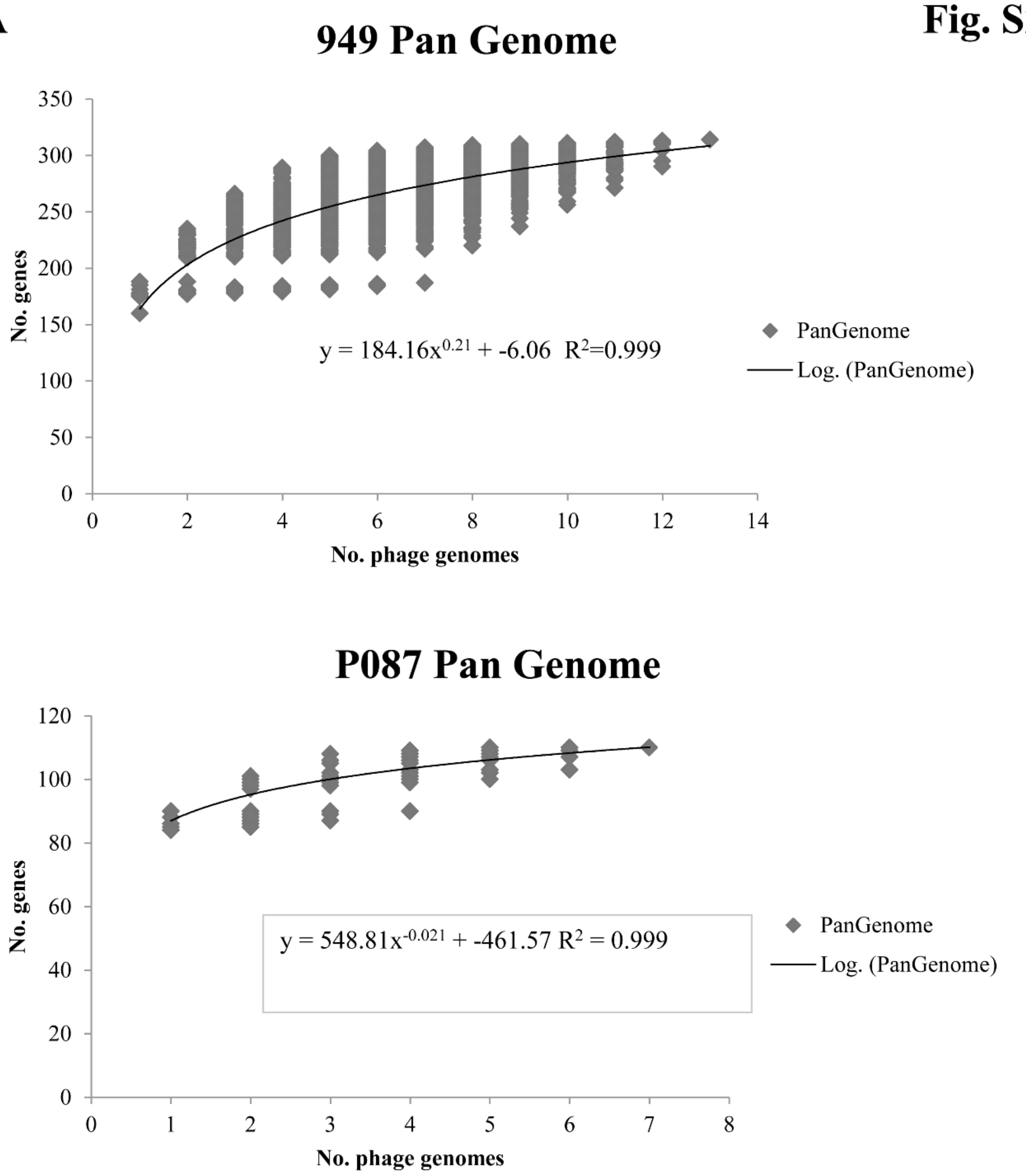

Figure S2. Pan-genome analysis of the sequenced members of the 949 (**A**) and P087 (**B**) phage groups using the PGAP pipeline. The pan-genomes of both phage groups appear closed having exponential values (x^n) of <0.5.

© 2017 by the authors. Licensee MDPI, Basel, Switzerland. This article is an open access article distributed under the terms and conditions of the Creative Commons by Attribution (CC-BY) license (http://creativecommons.org/licenses/by/4.0/).
